# Who participates in internet-based worksite weight loss programs?

**DOI:** 10.1186/1471-2458-11-709

**Published:** 2011-09-20

**Authors:** Wen You, Fabio A Almeida, Jamie M Zoellner, Jennie L Hill, Courtney A Pinard, Kacie C Allen, Russell E Glasgow, Laura A Linnan, Paul A Estabrooks

**Affiliations:** 1 Virginia Polytechnic Institute and State University, Dept of Agricultural and Applied Economics, Blacksburg, Virginia, USA; 2 Virginia Polytechnic Institute and State University, Dept of Human Nutrition Foods, & Exercise, Blacksburg, Virginia, USA; 3 Gretchen Swanson Center for Human Nutrition, Omaha Nebraska, USA; 4 Dissemination and Implementation Science Division of Cancer Control and Population Sciences, NCI, Rockville, Maryland, USA; 5 University of North Carolina, Dept of Health Behavior and Health Education, Chapel Hill, North Carolina, USA

## Abstract

**Background:**

The reach and representativeness are seldom examined in worksite weight loss studies. This paper describes and illustrates a method for directly assessing the reach and representativeness of a internet-based worksite weight loss program.

**Methods:**

A brief health survey (BHS) was administered, between January 2008 and November 2009, to employees at 19 worksites in Southwest Virginia. The BHS included demographic, behavioral, and health questions. All employees were blinded to the existence of a future weight loss program until the completion of the BHS.

**Results:**

The BHS has a participation rate of 66 percent and the subsequent weight loss program has a participation rate of 30 percent. Employees from higher income households, with higher education levels and health literacy proficiency were significantly more likely to participate in the program (p's < .01).

**Conclusions:**

Worksite weight loss programs should include targeted marketing strategies to engage employees with lower income, education, and health literacy.

## Background

A recent systematic review on the effectiveness of worksite nutrition and physical activity programs for reducing weight in overweight and obese employees found a consistent, but modest, effect of interventions on reductions in body weight (i.e., -2.8 pounds) and body mass index (BMI; i.e., -0.5 BMI) when compared to untreated controls [1]. These modest results could have a large public health impact if the intervention strategies could reach a large and representative or high risk sample of employees since the majority of adults spend a large amount of waking hours at work [2]. Unfortunately, few worksite health promotion studies reported on reach, [3] defined as the proportion of the eligible employee population that participated in a program and the representativeness of program participants compared to the eligible population [4]. Specifically, a review of worksite health prevention and intervention programs documented that only 25% of studies reported the proportion of eligible employees that agreed to participate in the study and only 9% reported on representativeness [3].

The current state of worksite intervention studies led the U.S. Preventive Services Task Force to conclude that there is a significant gap related to understanding the characteristics of the employee population that participates [1]. Because of this gap, it is unclear if those who could benefit most from a worksite intervention are as likely to participate as those who may already be making more healthful lifestyle choices [3,5]. This gap is also recognized in the general preventive care intervention research and several calls for better methods for reporting on external validity issues have been made [6-9] Understanding program reach not only would aid in filling the need of examining external validity of the program, but also would provide much needed information for determining whether or not the critical subgroups of employees are actually participating in the program. The results will also inform targeted marketing and recruitment efforts.

However, the documentation of reach and representativeness is predicated on knowing the proportion of eligible employees and having a clear picture of the demographic and behavioral characteristics of the total employee population, and this information has proven difficult to obtain. [e.g., [3,10-12]] Therefore the purpose of this paper is to describe and illustrate a method for directly assessing the reach and representativeness of participants in a worksite weight loss study that focused on changes in physical activity and nutrition.

## Methods

The data presented in this paper were collected as part of a two-group, cluster randomized controlled trial to investigate the reach and effectiveness of individually targeted, computer mediated worksite weight loss programs. The program comprises of emails with physical activity and diet messages that are tailored to the individual and includes small monetary incentives for weight-loss. Prior to program initiation a brief health survey (BHS) was administered to as many employees as possible at each participating worksite to determine eligibility, and evaluate reach and potential participation predictors. We administered the BHS between January 2008 and November 2009 with a goal to achieve a 70% completion rate prior to implementing the worksite weight loss intervention study. The analyses were completed in December 2009.

All employees with the exception of organizational decision-makers (i.e. CEOs and Human Resource Directors) were blinded to the existence of a future weight loss program to be delivered at their worksite, and as such employees' responses were unlikely to be biased by that knowledge. The BHS was introduced to the general employee population as a survey study to provide feedback on areas that the workplace health promotion efforts could focus on. The BHS was available in paper-and-pencil and web-based formats, and was delivered to all employees four weeks prior to the weight loss program being initiated. Employees had two weeks to complete the survey. All participating worksites allowed employees to complete the BHS during work hours. While it is noted in the literature that to achieve a 50 percent employee response rate to health risk surveys incentives of approximately $40 per employee are necessary when tied with strong organizational communication;[13] providing such incentives was beyond the budget of the project. As a result, a lottery system was installed to encourage the survey participation. Any BHS participant could submit his/her name for a $250 prize drawing at each worksite. This study was approved by the Virginia Tech Institutional Review Board (protocol #07-296).

### Worksites and Individual Employees Sample

Worksite eligibility criteria include internet access at the worksite and employing between 100 and 600 workers at that site. A total of 33 worksites were contacted. Nineteen agreed to participate in the study. Those that declined invitation did not differ from those that agreed on worksite size, location, or industry. Worksites that agreed to participate in the study included 3 governmental agencies, 5 professional groups, 3 medical facilities, 4 manufacturing and distribution centers, and 4 municipalities (Table 1). Worksites were primarily in urban areas (n = 16) with a small number from rural settings (n = 3).

**Table 1:** Participation Rates By Worksite

		BHS		
Worksite	Number of Employees	Completion Rate	% Eligible among those completed the BHS (BMI > = 25)	Program Participation Rate among Eligible Employees
***Governmental Agencies***				
Worksite 1	100	95%	79%	49%
Worksite 2	315	63%	71%	33%
Worksite 3	276	63%	83%	17%

***Professional Groups***				
Worksite 4	589	49%	73%	22%
Worksite 5	435	61%	50%	23%
Worksite 6	305	30%	69%	19%
Worksite 7	238	67%	81%	17%
Worksite 8	253	77%	75%	23%

***Medical Facilities***				
Worksite 9	246	83%	72%	47%
Worksite 10	291	79%	80%	28%
Worksite 11	477	70%	70%	19%

***Manufacturing and Distribution Centers***				
Worksite 12	197	72%	77%	23%
Worksite 13	243	58%	67%	21%
Worksite 14	353	61%	72%	18%
Worksite 15	297	71%	73%	27%

***Municipalities***				
Worksite 16	350	58%	72%	30%
Worksite 17	219	80%	74%	38%
Worksite 18	185	58%	92%	20%
Worksite 19	206	54%	86%	20%

Average	293	66%	75%	26%
Median	276	63%	73%	23%
Minimum	100	30%	50%	17%
Maximum	589	95%	92%	49%

Note: BHS - Brief Health Survey; BMI - Body Mass Index.

All employees were eligible to participate in the BHS. Employee eligibility criteria for the subsequent weight loss program include a BMI > = 25, regular employment status (e.g., temporary employees were excluded), and access to the internet. Therefore only those employees who were eligible to participate in the weight loss program are included in the analysis of this paper to determine weight loss program participant representativeness to the eligible population. The final analysis included 2,055 participants across 19 worksites ranging in size from 33-95 program participants and 100-589 total employees.

### Brief Health Survey Data

As the primary outcome measure, we created a dummy variable indicating whether an employee enrolled in the subsequent weight loss program or not. Key information collected through the BHS includes: healthy eating,[14] physical activity,[15] health status,[16] health literacy,[17] internet and e-mail use proficiency, self-efficacy,[18] response-efficacy,[19] and demographic variables (i.e., age, gender, race/ethnicity, number of children in the household, household income, and education). We also controlled in our model other factors such as self-reported height and weight, smoking status, and existence of comorbid conditions. Height and weight were used to compute BMI. Smoking status was assessed using a single-item question: "Do you currently smoke?" Comorbid health conditions (arthritis, asthma, depression, diabetes, heart disease, high blood pressure, high cholesterol, obesity, and none of the above) were assessed using a single-item question which respondents checked all conditions that have been diagnosed by a doctor.

### Reach and Representativeness Assessment

The initial reach metric calculated was the proportion of eligible employees (i.e., BMI > = 25) who enrolled in the weight loss program. The numerator for this analysis included all employees who enrolled in the study. The denominator used in this calculation was computed by multiplying the proportion of employees that responded to the BHS with a BMI greater than or equal to 25 by the total number of employees at the worksite to extrapolate to all eligible employees rather than just those who completed the BHS. Representativeness was calculated by comparing those eligible employees that participated in the program to eligible employees that did not participate in the program, but completed the BHS. In addition, there were a significant number of employees across worksites (n = 398) that participated in the program but did not complete the BHS. Thus we also compared these participants to those that completed the survey. This comparison allowed us to determine if those employees with a BMI ≥ 25 who responded to the BHS were representative of those who did not.

### Weight Loss Program Reach Prediction

We used a multi-level mixed effect logit model treating the employees as nested within worksite, which is equivalent to estimating random intercept models assuming the unobserved worksite-specific effects are not correlated with predictors in the model. The model allows us to apply the study results to general worksite populations. The variety of worksites in the program, shown in Table 1, supports the population inference as well.

## Results

### Reach and Representativeness Assessment

The BHS and the weight loss program reach rates are presented in Table 1. Across the 19 worksites enrolled in the study, the BHS participation rate on average is 66% with a median of 63% and ranged from 30% to 95%. For the weight loss program, the participation rate among the entire potentially eligible population ranged from 17% to 49% with a mean of 26% and a median of 23%.

Table 2 presents the representativeness assessment results. We used the BHS data to estimate reach therefore our sample is limited to the 2,055 individuals who completed the BHS. Among this sample, 610 employees enrolled in the weight loss program. The average participant was about 45 years old, with 59% being female and 72% being Caucasian, 25% African American, and 2.2% Hispanic. In general, participants were educated with 50% having college or professional/graduate degrees and 61% reported annual household earnings of $50,000 or more (Table 2).

**Table 2:** Representativeness of Brief Health Survey (BHS) and the Weight Loss Program

	Reach Study Sample (n = 2,055)	BHS & Program Participants (n = 610)	BHS Participants Program Nonparticipants (n = 1,445)	BHS Nonparticipants Program Participants (n = 398)	Group Mean Test p-value	Group Mean Test p-value	Group Mean Test p-value
	
Variables	Mean (1)	Mean (2)	Mean (3)	Mean (4)	Mean(2) = Mean(3)	Mean(2) = Mean(4)	Mean(1) = Mean(4)
Age	45.4	46.6	44.8	45.1	0.001***	0.034	0.706
Female, %	59.1	74.9	52.5	74.6	0.000***	0.916	0.000***
Hispanic origin, %	2.2	1.5	2.5	4.5	0.112	0.008***	0.007***
Race, %							
Caucasian	71.7	79.7	68.3	69.3	0.000***	0.000***	0.347
African American	24.6	18.0	27.3	25.1	0.000***	0.008***	0.815
Asian	1.2	0.3	1.5	1.3	0.003***	0.126	0.881
Other	2.6	2.0	2.8	4.3	0.222	0.048	0.064
Education, %							
Less than high school	2.0	0.3	2.7	1.5	0.000***	0.072	0.516
High school graduate	17.2	13.1	19.9	12.8	0.008***	0.890	0.032
Some college	30.3	33.9	28.7	29.9	0.021	0.178	0.884
College graduate	34.1	36.4	33.1	37.2	0.160	0.799	0.238
Post graduate/professional	16.4	16.2	16.5	18.6	0.862	0.337	0.295
Annual household Income, %							
Less than $15,000	2.0	0.3	2.7	2.3	0.000***	0.014	0.731
$15,000-$29,999	12.5	8.2	14.3	11.8	0.000***	0.066	0.719
$30,000-$49,999	24.1	25.9	23.4	22.6	0.231	0.232	0.514
$50,000-$99,999	41.7	43.9	40.8	38.9	0.185	0.116	0.306
More than $100,000	19.7	21.6	18.9	24.4	0.162	0.316	0.035
Have at least one child, %	50.8	49.7	51.3	56.3	0.506	0.040	0.045

Note: *** p < 0.01.

There were a total of 398 employees who did not complete the BHS but enrolled in the program. Those individuals were not in the program reach predication model analysis since their BHS data was missing. We compared their characteristics with the 610 program participants who completed BHS to assess the representativeness of our BHS sample. The seventh column of Table 2 presents the test results (i.e., comparing Mean(2) and Mean(4)). Majority of comparisons did not show statistically significant differences except for race/ethnicity which shows a relatively good representability of our BHS sample. However, those program participants who did not participate in the BHS on average were more likely to be Hispanic, or African American, and less likely to be Caucasian (p's < .01). To further assess the BHS representativeness, we compare those BHS participants with those 398 BHS nonparticipants. Results are shown in the last column of Table 2 (i.e., comparing Mean(1) and Mean(4)). It further confirms that our BHS sample is mostly representative except that female employees and those of Hispanic origin are less likely to fill out the BHS survey.

The results of the comparison test to assess the program participant representativeness are shown in the sixth column of Table 2 (i.e., comparing Mean(2) and Mean(3)). Statistically significant differences were detected comparing age, gender, proportion of Caucasian, African American, Asian, education level lower than some college, and income level lower than $30,000/yr.

### Reach Prediction Model

Table 3 provides the variable descriptions and summary statistics for variables of interest. Across worksites, weight loss program participation rate was 30% among those eligible employees who completed the BHS. The participation rate ranges across worksites between 20-50%. The participants reported relatively healthy eating habits, 5.93 (on a 14-pt scale with lower scores reflect more healthful eating patterns), but were generally inactive (1.66 on the 3-pt scale where 3 equals meeting recommended guidelines). There is wider variability in healthy eating habits than physical activity. Eighteen percent were current smokers and 56% reported at least one chronic health condition. The average BMI was 32, with a range of 25-64. The average health literacy score was also high (13 on the 15-pt scale). The 19 worksites in this study exhibit similar patterns overall but have sizable variations in terms of BMI and healthy eating behavior (with *SD *> 0.5).

**Table 3:** Variable Descriptions and Summary Statistics across Sample and Worksites

		Individual Level		Worksite Level	
		
N = 2,055	Description	Mean (SD)	Range	Mean (SD)	Range
*Outcome Variable*					
					
Participation	Dummy (= 1 if participate in the program; = 0 if not)	0.30 (0.46)	0-1	0.30 (0.08)	0.2-0.5
					
*Independent Variables*					
					
BMI	Body mass index score (kg/m^2^)	31.7 (5.75)	25.0-63.7	31.7 (1.27)	29.1-33.9
Physical Activity	Rank (= 1 inactive; = 2 MSR or MCV; = 3 Meeting recommendations)	1.66 (0.78)	1-3	1.66 (0.14)	1.5-2.0
Healthy Eating	Healthy eating scores. Lower scores means healthier eating habits	5.93 (2.52)	0-14	5.93 (0.58)	4.9-7.0
Overall Health Status	Ranked self-reported health status (1 excellent to 5 poor)	2.87 (0.83)	1-5	2.87 (0.16)	2.6-3.1
Comorbid Conditions	Dummy (1 if has at least one comorbidity; = 0 if has no other health conditions other than obesity)	0.56 (0.50)	0-1	0.56 (0.07)	0.4-0.7
Smoking	Dummy (= 1 if current smoker; = 0 otherwise)	0.18 (0.39)	0-1	0.18 (0.08)	0.1-0.3
Health Literacy	Health literacy scores. Higher scores means better health literacy.	13.25 (1.91)	3-15	13.25 (0.44)	12.0-14.0
Internet Use	Self efficacy in internet use. Higher scores means higher efficacy.	3.59 (1.61)	1-5	3.59 (0.22)	3.2-4.2
Email Use	Self efficacy in email use. Higher scores means higher efficacy.	3.60 (1.64)	1-5	3.6 (0.25)	3.1-4.2

Note: SD - standard deviation.

The multi-level mixed effect logit model was significant (Wald Test, χ^2 ^= 172.35, *p *< .000) and the Likelihood Ratio test was significant (5.81, p < .01) indicating that the model was more appropriate than the standard logit model (Table 4). The odds of a female employee participating in the program are 3.3 times higher than their male counterparts while those African American and Asian employees are less likely to participate in the program compared to Caucasian employees. For a one category increase in household income and education level, odds of participation increased by 16% and 12% respectively. One unit score increase of BMI will increase the odds of participation by about 3%. The odds of participation are 1.56 times higher for non-smokers and 1.23 times higher for employees reporting existing health conditions. An increase in health literacy score by one unit resulted in an 8% increase in the odds of program participation. Physical activity level, healthy eating behaviors, self-reported overall health status, other race compared to Caucasian, and Hispanic origin are not significant predictors.

**Table 4:** Program Participation Multi-level Mixed Effect Logit Model Results

	OR	SE	p-value	CI(95%)
BMI	1.03***	0.01	0.002	1.01-1.05
Physical Activity	0.89	0.07	0.130	0.77-1.03
Healthy Eating	1.01	0.02	0.693	0.97-1.05
Overall Health Status	1.01	0.07	0.848	0.88-1.17
Comorbid Health Conditions	1.23*	0.14	0.075	0.98-1.53
Smoking	0.64***	0.09	0.002	0.48-0.85
Health Literacy	1.08**	0.03	0.013	1.02-1.15
Female	3.26***	0.40	0.000	2.56-4.13
Age	1.01**	0.005	0.031	1.00-1.02
Race (Caucasian is the base)				
African American	0.51***	0.07	0.000	0.38-0.67
Asian	0.25*	0.19	0.067	0.06-1.10
Other	0.79	0.28	0.505	0.39-1.60
Hispanic	0.61	0.25	0.225	0.27-1.36
Education	1.12**	0.07	0.069	0.99-1.26
Income	1.16**	0.07	0.015	1.03-1.31
Have at least one child	1.08	0.12	0.452	0.88-1.34

Wald chi2	172.35		0.000	
LR (mixed effect logit vs. normal logit)	5.81		0.008	

Note: OR - odds ratio; SE - standard errors; CI - confidence intervals;

* p < 0.1, ** p < 0.05, *** p < 0.01.

## Discussion and Conclusion

This paper describes an initial attempt at addressing the paucity in the research literature on the reach of worksite-based weight loss programs. Consistent with the literature, we found that older Caucasian women were more likely to participate in worksite weight loss programs [5,12]. Of note, even though the weight loss programs were internet-based more women than men as well as older people were willing to participate suggesting that digital divide stereotypes may not apply in this context [20]. Our study also adds to the literature and suggests that participants are more likely to have a chronic condition, higher BMI, and higher health literacy than the overweight and obese employees that choose not to participate.

A primary goal of this paper was to present a method to assess reach and representativeness in the context of worksite weight loss programs. While it would have been ideal to achieve a 100% completion rate of the BHS, the average completion rate of approximately two thirds of the employee population reflects a strong response rate relative to other worksite health risk appraisal completion data [13]. This suggests that a lottery incentive system, when coupled with strong organizational communication strategies, could be as effective as individual level incentives. Further, our study also provides researchers a more cost effective method of achieving a greater than 50 percent BHS response rate.

We also found that African American and Asian employees were about one-half and one-quarter less likely than Caucasians to participate, respectively. Similar to the need to use culturally sensitive strategies within weight loss programs for diverse employee populations,[21,22] our findings suggest the need to also develop culturally-tailored recruitment tactics and materials. For example, African Americans may be less likely to perceive themselves as overweight and more likely to associate attractiveness and health status with heavier body size when contrasted with Caucasians [23,24]. For these reasons, programs that focus on outcomes aside from weight loss such as obesity-related co-morbid conditions or improved job performance and satisfaction may promote higher participation among African Americans. Similarly, employees with lower health literacy were found to be less likely to participate in the program which confirms the important implications of health literacy status in the context of recruiting and retaining participants [25,26]. Our study directly assessed health literacy as a characteristic impacting enrollment which no known study has done before.

Limitations of this study include varying participation rates in surveys and an average BHS completion rate of approximately 66%. Although conducting a thorough examination of potential non-response bias was beyond the scope of this study,[27] we did calculate adjusted reach estimates to account for these differences and provide comparison tests to assess BHS representativeness (Table 2). In contrast study strengths include a diverse set of worksites, a range of demographic, behavioral, and health literacy indicators; the study of both survey and program participation; and adjustments for clustering of employees within worksites.

The use of a BHS to provide information on employee characteristics and eligibility provides an opportunity to determine more accurate indicators of reach and representativeness. As reach and representativeness have received limited attention in worksite health promotion research,[3] it is necessary to persist in collection and reporting of these types of data. The approach utilized in our study appears to be feasible and appropriate for use in future research.

## Competing interests

The authors declare that they have no competing interests.

## Authors' contributions

WY participated in the design of the study, performed the statistical analysis and drafted the manuscript. FA participated in the study design, lead and manage the acquisition of data, and was involved in drafting the manuscript. JZ, JH, CP, and KA participated in interpretation of results and were involved in drafting of the manuscript. RG and LL contributed in drafting the manuscript and the design of the study. PE conceived of the study, lead the study design, contributed in statistical analysis and results interpretation, coordinated and participated in the drafting of the manuscript. All authors read and approved the final manuscript.

## Pre-publication history

The pre-publication history for this paper can be accessed here:

http://www.biomedcentral.com/1471-2458/11/709/prepub
